# Implications of Renal Denervation Therapy in Patients with Sleep Apnea

**DOI:** 10.1155/2015/408574

**Published:** 2015-09-30

**Authors:** Fernando Jaén-Águila, José Antonio Vargas-Hitos, Juan Diego Mediavilla-García

**Affiliations:** Cardiovascular Risk Unit, Department of Internal Medicine, University Hospital Complex City of Granada, Avenida Fuerzas Armadas 2, 18014 Granada, Spain

## Abstract

Obstructive sleep apnea (OSA) syndrome is a prevalent condition characterized by repeated episodes of obstruction of the upper airway, leading to intermittent hypoxemia and important endothelial and anatomical dysfunctions that cause cardiovascular and cerebrovascular disease. The finding of the relationship between OSA and hypertension, especially resistant hypertension (RHT), has increased the interest in therapeutic strategies that affect renal sympathetic activity in these patients. The observational studies published until now demonstrated that renal denervation therapy can reduce the severity of OSA syndrome. Renal sympathetic denervation (RDN) could be a future therapeutic possibility for conditions other than RHT, such as atrial fibrillation, heart failure, obesity, and OSA syndrome, where renal sympathetic system plays an important physiological role. The aim of this review was to elucidate the implications of renal sympathetic activity in OSA syndrome.

## 1. Introduction

The apnea-hypopnea sleep (AHS) syndrome was firstly described in 1976 by Guilleminault et al. [1]. It affects 3–7% of the general population and is characterized by repeated episodes of obstruction of the upper airway during sleep.

The apnea-hypopnea index (AHI) is used to measure the severity of obstructive apnea. Diagnosis is defined as an AHI > 5 accompanied by disease-related symptoms [2].

OSA syndrome and its relationship with HT have been well established [3]. An increase in the AHI implies an increased risk of developing HT [4]. The factors involved in this cardiovascular issue are endothelial dysfunction and systemic inflammation, which lead to activation of the sympathetic tone. Excess sympathetic tone plays a decisive role in the development of RHT [5]. It is an independent risk factor for cardiovascular risk, which is responsible for ischemic heart disease, atrial fibrillation, heart failure, stroke, and sudden death [6]. Two-thirds of the patients with acute ischemic stroke develop OSA syndrome. The presence of OSA syndrome in patients who suffered from cerebrovascular events determines impairment of cognitive function during the acute and subacute phases of stroke [7].

HT is a major public health issue worldwide, not only because of its high prevalence (30–45% of the general population, reaching 60% in the elderly), but also because of the impact on cardiovascular morbidity and mortality. Population studies have reported that one-third of the hypertensive patients develop apnea-hypopnea sleep (AHS) syndrome and that 40% of patients with AHS syndrome are hypertensive [4, 8].

It has been observed that OSA syndrome is present in 75% of patients diagnosed with RHT; nevertheless, its prevalence is much lower (38%) in hypertensive patients with controlled blood pressure (BP) [9]. Cardiovascular risk was found to be significantly higher in patients with RHT than in those without RHT [10]; this association opens the possibility that OSA syndrome and HT share similar physiopathological mechanisms contributing to both pathological processes. Recent expert information recommends the treatment of OSA syndrome as part of the management of patients with RHT [11–13].

The increase of sympathetic activity is involved in the development, management, and the evolution of the hypertensive state of patients with OSA syndrome. It implies that sympathetic deactivation can be seen as a goal of treatment. Small observational studies published so far reported that RDN can decrease the severity of OSA syndrome. RDN therapy is having an important role in the treatment of other diseases apart from HT; in fact, beneficial effects of RDN therapy in diseases other than HT where the renal sympathetic system plays an important role have been reported, such as atrial fibrillation, heart failure, obesity, or diabetes. The aim of this review was to elucidate the implication of renal sympathetic activity on the OSA syndrome.

## 2. Physiopathological Factors of OSA Syndrome

Many physiopathological factors have been described involved in the development of AHS syndrome. Some of the factors that promote the collapse of the upper airway are the narrowing of this airway, excessive loss of muscle tone, and defective upper airway protective reflexes mediated by overstimulation of sympathetic nervous system [14–16].

**Figure 1:**
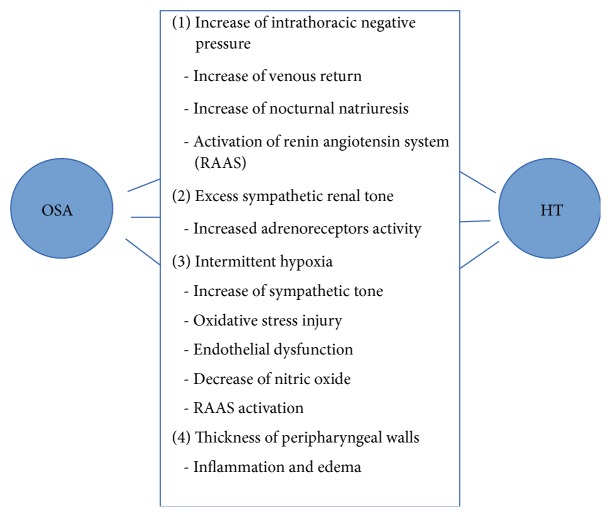
Relationship between OSA syndrome and arterial hypertension (HT).

The neurophysiological factors involved in the development of HT in patients with AHS syndrome are interacting with each other [17]. Increased sympathetic tone implies the following physiological changes (Figure 1):

(1) Intermittent hypoxia due to apnea and hypopnea triggers an excess of sympathetic activity by the activation of the carotid chemoreceptors; it leads to direct vasoconstriction and the subsequent stimulation of the renin-angiotensin-aldosterone system (RAAS) as well as increased levels of endothelin and angiotensin II. Activation of the renin-angiotensin axis produces fluid retention due to sodium reabsorption; it seems to lead to edema in the peripharyngeal walls, which predisposes to upper airway obstruction [18, 19].
(2) The increase in the sympathetic tone in patients with OSA syndrome produces renal activation of the autonomic nervous system. The kidneys are connected to the brain by afferent and efferent pathways. Hyperactivity of the autonomic nervous system stimulates renin release from the juxtaglomerular apparatus, then activating *β*1 adrenoreceptors; this increases the circulating volume when sodium retention increases, and renal blood flow decreases through the *α*1 adrenoreceptors.
 Renal afferent activation determines an increase of the sympathetic activity in the central nervous system (CNS), involving the vascular system, heart, and the other peripheral organs, leading to HT and its degree of severity [20].
(3) Sympathetic hyperactivity reduces the dilating effect of the upper airway muscles mediated by the genioglossal nerve and predisposes to pharyngeal obstruction [21]. Excess of sympathetic tone increases pharyngeal wall thickness and favors peripharyngeal fluid accumulation promoting the development of OSA syndrome, with recurrent episodes of hypoxia, sleep fragmentation, and the subsequent increase of the sympathetic tone [22].
(4) The physiological stimuli associated with apnea produce the formation of endogenous vasoactive substances and decrease the levels of nitric oxide, a potent vasodilator. It has been demonstrated that the use of CPAP during the night increases the circulatory levels of nitric oxide. Ischemic and reperfusion events associated with apnea lead to endothelial injury [23].

## 3. Results of the Major Clinical Studies in Patients with OSA Syndrome Undergoing Renal Sympathetic Denervation

Symplicity HTN-1, HTN-2, and HTN-3 trials are the most relevant studies concerning the clinical use of RDN therapy [30–32]. The initial studies demonstrated a significant decrease of BP levels at 3 years in patients with RHT.

Nevertheless, the Symplicity HTN-3 study which enrolled 535 randomized patients with a 6-month follow-up was not able to confirm the results previously obtained. There was no significant between-group difference in the change in office blood pressure at 6 months. This finding has questioned its efficacy. However, the responses with regard to systolic and diastolic blood pressure were significantly greater in the denervation group than in the sham-procedure group. It seems to be demonstrated that RDN therapy reduces renal sympathetic secretion and leads to a systemic decrease in sympathetic tone.

Some small studies have demonstrated a significant decrease of AHI in patients with OSA syndrome after undergoing RDN; this can explain the relationship between HT, OSA syndrome, and excess sympathetic tone. RDN has the potential effect of decreasing sympathetic overactivity in patients with OSA syndrome [33].

The first studies demonstrating this relationship were carried out in animal models. RDN decreased BP rises and the incidence of secondary arrhythmias, during postapneic periods in OSA syndrome models. This decreased the susceptibility of these animal models to develop atrial fibrillation [34]. Furthermore, these effects seemed to be independent of the decreases found in BP levels, which gave more relevance to the role of the central sympathetic secretion.

A recent meta-analysis published by Shantha and Pancholy [24] included 5 relevant clinical studies [25–29] in humans (Table 1) with a total of 49 patients studied.

Three of these studies were carried out in Europe, where the RDN technique is more accepted. Of the five studies, that of Witkowski et al. evaluated specifically the role of RDN in the AHI, BP, and glycemic control. Although the number of patients involved in the study was small (*n* = 10), it showed relevant results. AHI was measured in the 10 patients by polysomnography, before and 6 months after undergoing RDN. The authors reported a decrease in the severity of OSA syndrome in the patients after RDN, although without statistical significance due to the small size of the sample. The authors also reported that RDN therapy significantly decreased BP levels and improved the glycemic control of patients [28].

Zhao et al. [29] compared the response a total of 31 patients with OSA syndrome, 16 of them undergoing CPAP treatment and the other 15 treated with RDN.

The authors concluded that both CPAP and RDN treatments decreased OSA severity in the patients. They reported that the efficacy of CPAP treatment was higher in patients undergoing this therapy, since 6 months after treatments the AHI was lower in the patients treated with CPAP than in those who underwent RDN.

All 5 studies of the meta-analysis showed significant changes in BP after RDN, with a 6-month follow-up. The decrease in SBP was greater than in DBP. Furthermore, there were some evidences that the decrease was independent of the improvement in the severity of OSA found in the patients. The meta-analysis included 49 patients followed up during 6 months. The AHI was measured in all of them before and after RDN. The results demonstrated a reduction in AHI 6 months after RDN, as well as less nocturnal awakenings and improvement of nocturnal oxygen saturation.

## 4. Treatment of Sleep Apnea in Patients with Hypertension

Continuous positive airway pressure (CPAP), described by Sullivan et al. in 1981, is the treatment of choice for sleep apnea in hypertensive patients [35]. It improves the apnea episodes and prevents oxygen desaturation and the arousals (electroencephalographic awakenings), which results in a reduction in the morbidity and mortality of these patients [36].

Montesi et al. [37] carried out a systematic review and meta-analysis in 2012, which included 32 studies and a total of 2303 patients. The use of CPAP therapy was associated with a significant decrease in SBP, DBP, and mean BP values. BP decreases were mainly found in patients with higher daytime sleepiness, more severe OSA, and more compliance to CPAP therapy.

The mechanisms of the association between BP and daytime sleepiness could be related to the arousals, which occur at the end of the respiratory events, coinciding with repeated BP surges. Intermittent ischemic episodes increase BP by the activation of type I angiotensin II receptor. It seems that overactivation of the renin-angiotensin axis occurs independently of other factors [38]. RDN could have an important role in patients with higher sympathetic stimulation.

Although the hypotensive effect of CPAP seems to be moderate, decreases of 5 mm Hg in DBP reduce the risk of cerebrovascular accidents by 42% and all cardiovascular events by 31% [39].

Significant decrease of BP only 3 weeks after onset of CPAP treatment reinforces the importance of studying and treating OSA syndrome in patients with RHT.

## 5. Conclusions

CPAP is the treatment of choice in the management of patients with OSA; its use in the treatment of RHT is becoming increasingly more widespread. The important implications of morbidity in OSA syndrome make it necessary to seek new therapies that intervene in the physiological mechanisms related to cardiovascular events in these patients. Nowadays, it has been demonstrated that RDN improves the severity of OSA syndrome in patients with RHT. In this respect, RDN treatment could be considered in patients with excess sympathetic tone. Nevertheless, further clinical trial should be required before renal denervation can be applied to the study of OSA and other conditions such as obesity hypertension.

## Figures and Tables

**Table 1:** Major clinical studies carried out in patients with AHS syndrome undergoing RND therapy. Modified by Shantha and Pancholy [24].

Author	Year	Patients (*n*)	OSA patients	AHI pre-RDN	AHI post-RDN (6 months)
Damascelli et al. [25]	2013	24 RHT patients	2	63.3	26.5
Schmiedel et al. [26]	2013	40 RHT patients	16	25	17
Thakur et al. [27]	2013	21 RHT patients	6	21.1	10.5
Witkowski et al. [28]	2011	10 RHT patients, all with OSA	10 10	30.7	16.1
Zhao et al. [29]	2013	31 RHT patients	15	32	27

Resistant hypertension = RHT; obstructive sleep apnea = OSA; apnea-hypopnea index = AHI; renal sympathetic denervation = RDN; apnea-hypopnea sleep syndrome = AHS.
